# Supplementary material for: Direct observation of topological magnon polarons in a multiferroic material
Source: Nat Commun. 2023 Sep 29;14:6093. doi: 10.1038/s41467-023-41791-9 (PMC10541872; doi:10.1038/s41467-023-41791-9)
Supplement: Supplementary file 1 — Supplementary Information [file 41467_2023_41791_MOESM1_ESM.pdf]

Supplementary Information for

Direct observation of topological magnon polarons in a multiferroic material

S. Bao *et al.*

This PDF file includes:

Supplementary Figs. 1 to 4

Supplementary Tables 1 to 3

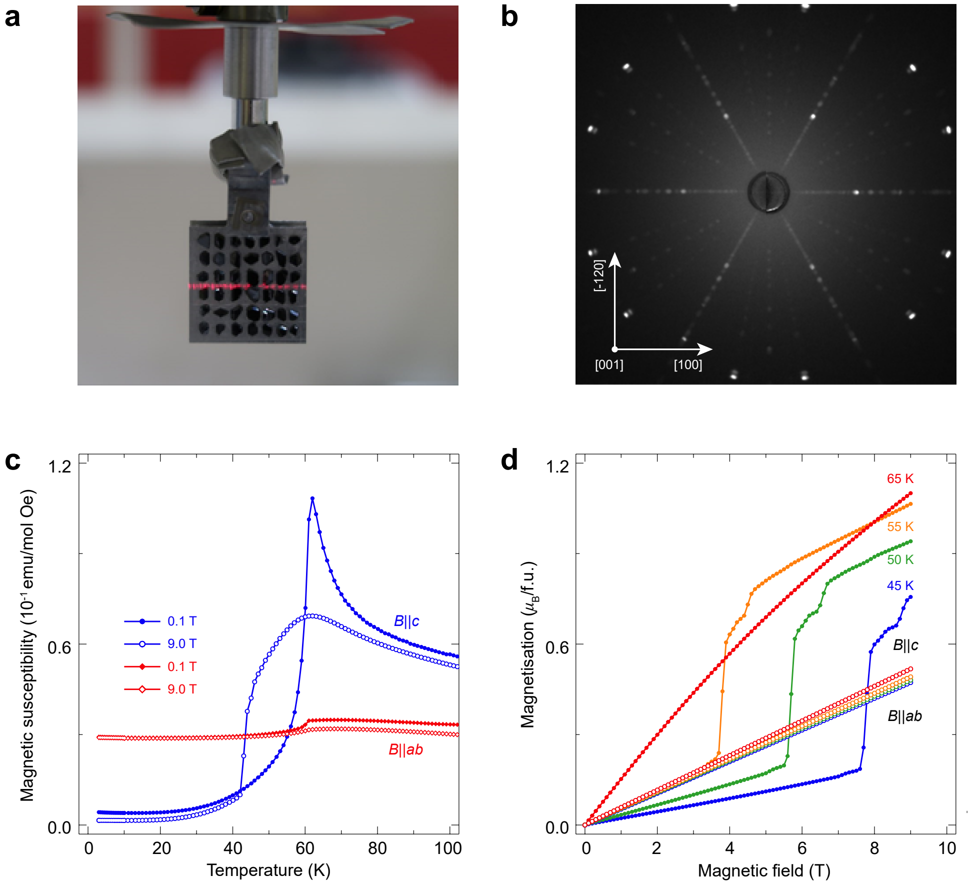

Supplementary Fig. 1. Single crystals of $Fe_2Mo_3O_8$ and characterisations. **a**, A photograph of the measured sample array. The 150 pieces of single crystals with a cleavage plane (001) were glued onto four plates, each of which has the dimensions of 26 × 26 × 0.3 mm^3. Each pair of plates was stuck back to back, and the two pairs of plates were assembled with aluminum nuts, resulting in a stacking thickness of 6.4 mm. Scattering data were collected by rotating the sample about the [-120] direction, and a negligible effect on neutron absorption due to sample rotation was observed in our measurements. **b**, Laue x-ray pattern of a single crystal with the beam along [001] direction. **c**, Temperature dependence of the magnetic susceptibility on a single crystal for the field applied along c axis (blue) and in a-b plane (red). Filled and empty symbols represent a field of 0.1 and 9.0 T, respectively. **d**, Magnetisation as a function of magnetic field at various temperatures for fields applied both in and out of plane. In **c**, it is found that a λ-type peak occurs around 60 K with a 0.1-T field applied along c axis, indicating the antiferromagnetic transition. However, at 9.0 T, that peak becomes broad which is due to the metamagnetic transition to

a ferrimagnetic state. While upon cooling, the antiferromagnetic state will be established at a lower temperature, evidenced by a sudden drop of the magnetisation around 40 K. The metamagnetic transition can also be observed by the dramatic increase in the isothermal magnetisation curves in **d**. For the field applied perpendicular to c axis, there is no obvious difference in the magnitude of susceptibility at 0.1 and 9.0 T, in contrast to those with field applied along c axis. Below the transition temperature, the susceptibility drops slightly to a constant rather than approaching zero as those with field applied along c axis. No any metamagnetic transition can be recognized up to 9 T, as can also be revealed by the linear relationship in the isothermal magnetisation curves in **d**. These results indicate $Fe_2Mo_3O_8$ has a collinear antiferromagnetic ground state with strong easy-axis anisotropy along c axis.

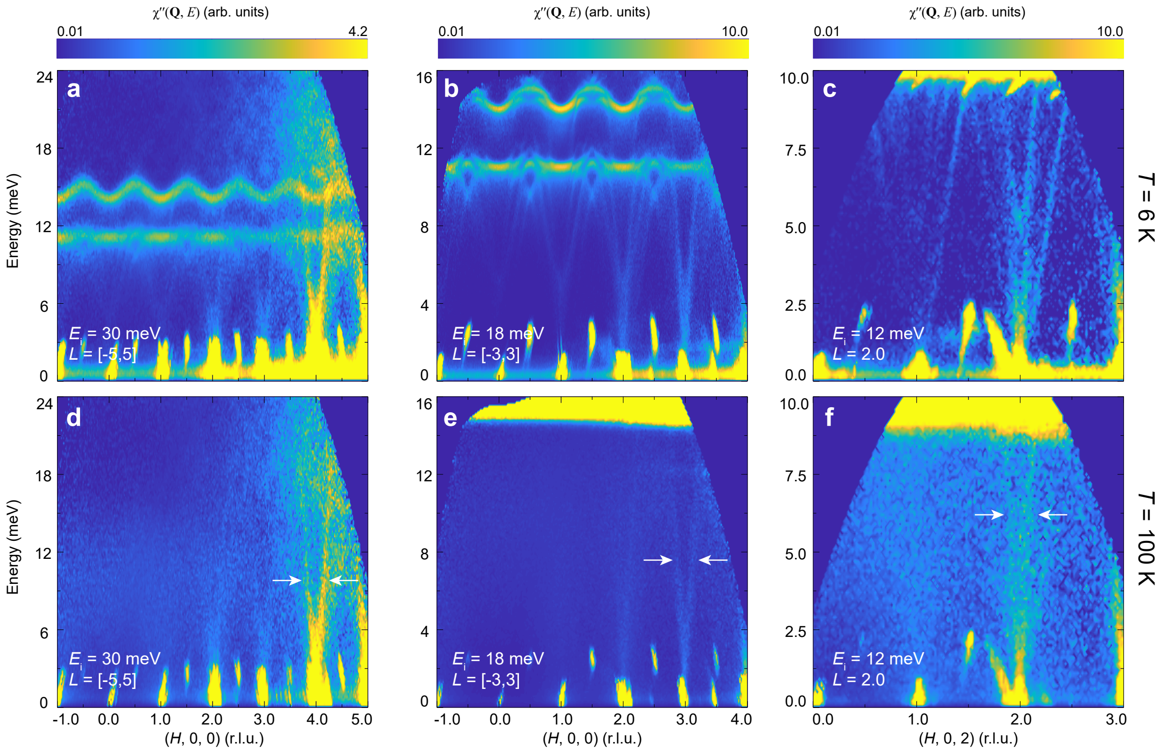

Supplementary Fig. 2. Additional INS results along [100] direction. a-c, Excitation spectra at $T = 6$ K, measured with $E_\mathrm{i} = 30$ meV (**a**), $E_\mathrm{i} = 18$ meV (**b**) and $E_\mathrm{i} = 12$ meV (**c**) on 4SEASONS. **d-f**, Same as in **a-c**, but measured at $T = 100$ K. Bright spots above and elongated signal near the elastic line are spurious peaks caused by saturation of the neutron detectors. There are a few points worth mentioning: (i) The intensities of the low-energy bands become stronger as the wavevector H increases, a feature expected for phonons. (ii) These acoustic phonon modes survive at large **Q**s but disappear at small **Q**s at 100 K. The reason may be that the spin components they obtain from magnon-phonon interaction, which dominate the scattering intensities at small **Q**s at 6 K, will disappear at 100 K due to the collapse of magnons. Meanwhile, the intrinsic structure factor for phonons at small **Q**s are relatively small. (iii) Since the data have been corrected by the magnetic form factor, intensities of the magnons at different zones remain almost constant. The reason that they become slightly weaker at very large H as shown in **a** may be that the magnetic form factor under dipole approximation will be invalid at large **Q**s, and the role of spin-orbit and spin-lattice coupling is ignored.

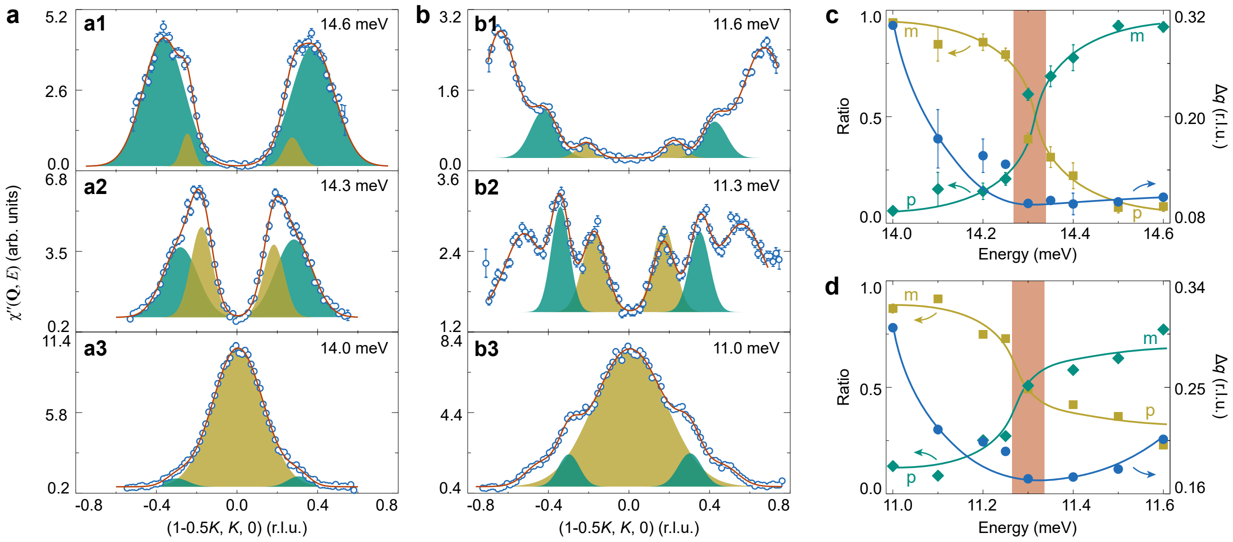

Supplementary Fig. 3. Spectral evolution of the magnon-polaron bands along [-120] direction. These results are obtained in the same way as those in Fig. 4g-j of the main text, but along [-120] direction.

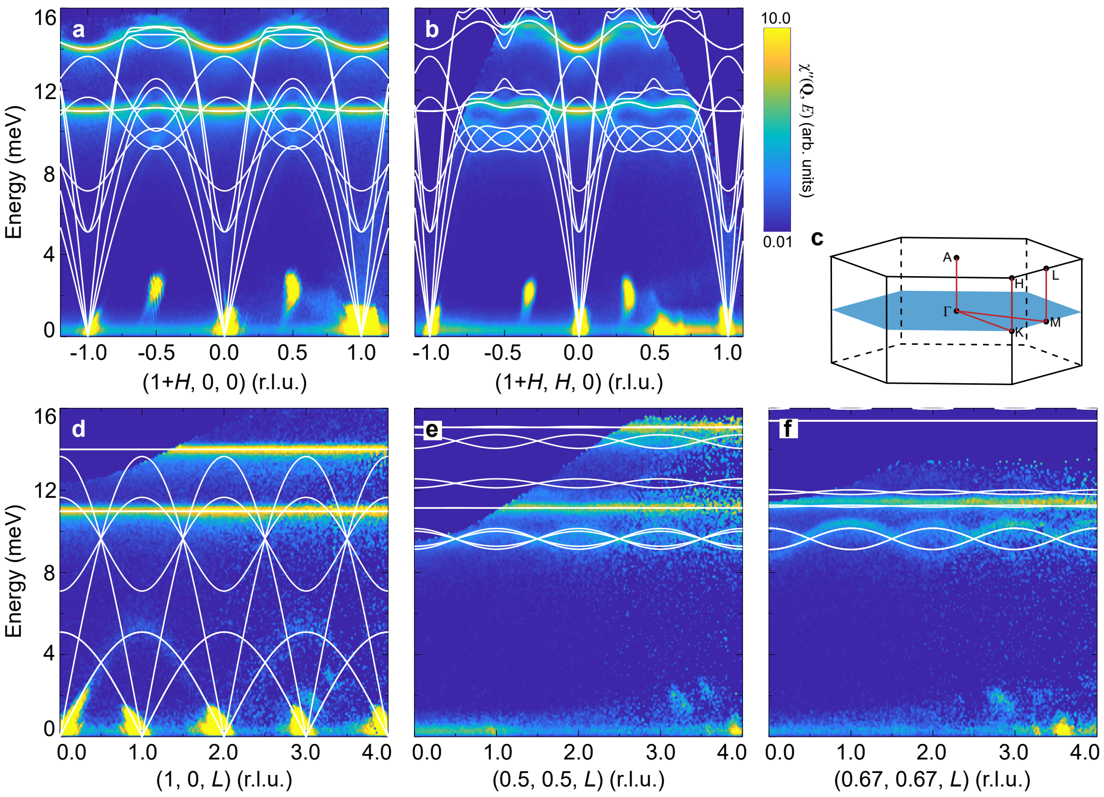

Supplementary Fig. 4. Comparisons between calculations using the three-dimensional model and the experimental excitation spectra at 6 K. **a**, **b** In-plane excitation spectra along [100] and [110] directions, respectively. **c**, Three-dimensional Brillouin zones with high-symmetry points and paths. **d**-**f**, Out-of-plane excitation spectra along [001] starting at Γ, M and K points, respectively. The experimental spectra in **a**, **b** and **d** are the same with those in Fig. 2a-c in the main text. In **d**-**f**, data on the negative L side have been symmetrised to the positive side to improve the statistics. For better display, these data are plotted in a logarithmic scale of intensity. All data were measured with $E_\mathrm{i} = 18$ meV on 4SEASONS. Solid lines on top of the experimental spectra are the results calculated using the three-dimensional model. The spring constants are listed in Supplementary Table 3. It is worth noting that one of the three acoustic bands with the lowest group velocity along the in-plane directions (**a**, **b**) corresponds to out-of-plane polarised phonons. This particular band is lower in energy than the magnons, suggesting the negligible hybridization through magnetoelastic coupling. The other two acoustic bands with in-plane polarisation are found to be hybridized with magnons through the DM-interaction-induced magnon-phonon coupling.

Supplementary Table 1. The integration range used to plot figures. The wavevectors for the integration are labelled in the first row of the table unless specified otherwise. The superscript $*$ represents the integration along [-$K, K, 0$] direction (the other in-plane orthogonal direction is changed to $[H, H, 0]$ accordingly), which is equivalent to $[H, 0, 0]$ direction.

Figures	$[H, 0, 0]$ (r.l.u.)	[-0.5$K, K, 0$] (r.l.u.)	$[0, 0, L]$ (r.l.u.)
Fig. 2a	/	[-0.06, 0.06]	[-3, 3]
Fig. 2b	/	[-0.55, 0.45]*	[-3, 3]
Fig. 2c	[0.95, 1.05]	[-0.06, 0.06]	/
Fig. 3a, d	/	[-0.06, 0.06]	[0.8, 1.2]
Fig. 3b, e	[0.95, 1.05]	[-0.06, 0.06]	/
Fig. 3c, f	[1.95, 2.05]	[-0.06, 0.06]	/
Fig. 4a-f	/	/	[-3, 3]
Fig. 4g, i	/	[-0.06, 0.06]	[-3, 3]
Supplementary Fig. 2a, d	/	[-0.06, 0.06]	[-5, 5]
Supplementary Fig. 2b, e	/	[-0.06, 0.06]	[-3, 3]
Supplementary Fig. 2c, f	/	[-0.06, 0.06]	[1.8, 2.2]
Supplementary Fig. 3a, b	[0.95, 1.05]	/	[-3, 3]
Supplementary Fig. 4a	/	[-0.06, 0.06]	[-3, 3]
Supplementary Fig. 4b	/	[-0.55, 0.45]*	[-3, 3]
Supplementary Fig. 4d	[0.95, 1.05]	[-0.06, 0.06]	/
Supplementary Fig. 4e	[0.7, 0.8]	[0.44, 0.56]	/
Supplementary Fig. 4f	[0.95, 1.05]	[0.61, 0.73]	/

Supplementary Table 2. Heisenberg couplings, magnetocrystalline anisotropy constants and DM interaction with the unit of meV, and spring constants with the unit of (meV)^2 for $Fe_2Mo_3O_8$ in the two-dimensional effective model.

J_1	$J_2^{\rm o}$	$J_2^{\rm t}$	J_3	$\Delta^{\rm o}$	$\Delta^{\rm t}$	D
0.5742	-0.06522	-0.01386	-0.2113	-3.005	-2.250	0.8
k_1/M	$k_2^{\rm o}/M$	$k_2^{\rm t}/M$	k_3/M	k_4/M	/	/
36.5	9.1	7.6	15.5	5.5	/	/

Supplementary Table 3. Spring constants of $Fe_2Mo_3O_8$ in the three-dimensional effective model. In the three-dimensional effective model, apart from the terms already present in the two-dimensional one (Supplementary Table 2), we include the interlayer couplings of the phonons while still keeping the magnons two-dimensional. This is because along c axis phonons have noticeable dispersions while magnons are rather flat. The interlayer longitudinal spring constants of phonons are considered up to the 3rd nearest order of interlayer bonds, denoted by $k_1^\perp$, $k_2^\perp$ and $k_3^\perp$. It is also found that with only the longitudinal components of the interlayer spring constants, the out-of-plane dispersions of the phonons cannot be reasonably reproduced, which indicates the transverse components of the interlayer spring constants must be considered. To reduce the tuning parameters and also keep the symmetry of the system, instead, we phenomenologically tune the distance d between the two honeycomb layers, which can effectively introduce transverse components to the interlayer dynamical matrix. The final phenomenological parameters of the phonon part are listed in this table with $d = 8.7973$ Å. The unit for these parameters is $(\text{meV})^2$. The parameters of the magnon part and the DM interaction are the same to those used in the two-dimensional effective model (Supplementary Table 2).

k_1/M	k_2^o/M	k_2^t/M	k_3/M	k_4/M	$k_1^\perp/M$	$k_2^\perp/M$	$k_3^\perp/M$
36.5	7.6	6.1	15.5	5.5	4.0	2.4	3.1
